# Osseous Tissue Formed from Transplanted Bone-Marrow

**Published:** 1882-05

**Authors:** 


### OSSEOUS TISSUE FORMED FROM TRANSPLANTED BONE-MARROW.

Professor Bruns, of Tubingen, reports (*Archiv fuer Klin. Chir.*, Bd. XXVI. Heft 3) the results of some experiments he has lately made on animals, with the object of determining whether portions of transplanted bone-marrow can give rise to the formation of deposits of true osseous structure. The professor states that the animals best suited for experiments of this kind are young dogs. A portion of the shaft of the femur or tibia is resected, and the marrow contained in this resected fragment is removed in an unbroken cylinder. Portions of this cylinder are then inserted into fresh wounds on the breast or back of the same animal, either into the subcutaneous fat or in a superficial part of the muscular layer. The wounds are then carefully closed by means of sutures.

The following changes, it is stated, take place in each instance of successful transplantation. A diffuse swelling is at once formed, which speedily begins to diminish, and is replaced about the fourteenth day by a movable nodule, in which bony tissue already exists in scattered foci. By the twenty-fourth day, foci have usually amalgamated into a single piece of bone. Microscopical examination proves that the nodule, in its early stages, is composed of osteoid tissue, cartilage, and newly formed osseous tissue, and that the fully developed hard mass consists of true bone.

These experiments, Professor Bruns asserts, prove that bone-marrow, completely separated from its connection with bone, and transplanted under the skin of the same animal, at a remote part of the body, may give rise to the formation of bone and cartilage. The swelling at the seat of transplantation ossifies in part directly and in part by the conversion of cartilage and osteoid tissue, into hard bone. The same process takes place in the formation of both the inner and outer callus after fracture ; and it may be assumed that bone is formed from the medulla in a way similar to that in which it is formed from the inner surface of the periosteum. It is held by Professor Bruns that in each instance the osteogenetic function is due to the same elements, namely, to osteoblast, which exist in the inner periosteal layer and are scattered among the elements of bone-marrow, particularly in young animals. Professor Waldeyer, of Stasburg, who has examined these specimens, agrees in the view of the part played by the osteoblast in the ossification of marrow, and is not disposed to admit any participation in this process of leucocytes of the marrow, wandering leucocytes from the blood, metamorphosed fat cells, or newly formed spindle-shaped connective tissue cells.—*London Med. Record.*

